# Mapping the potential aggregation values of ecotourism landscapes from stakeholder survey, structural equation modeling and GIS: Case study of Moc Chau site, Vietnam

**DOI:** 10.1371/journal.pone.0253908

**Published:** 2021-07-01

**Authors:** Tuan Anh Pham, Tam Minh Pham, Giang Thi Huong Dang, Doi Trong Nguyen, Quan Vu Viet Du

**Affiliations:** 1 Faculty of Social Sciences, Tay Bac University, Son La, Vietnam; 2 Faculty of Geography, VNU University of Science, Hanoi, Vietnam; 3 Faculty of Natural Science, Quy Nhon University, Binh Dinh, Vietnam; University of Defence in Belgrade, SERBIA

## Abstract

The primary aim of this study is to propose a potential landscape value assessment from different dimensions rather than the traditional approach of a composite indicator. The method used in this study is the combination of data collection from stakeholder survey, score measurement for landscape value dimensions using Structural Equation Modeling (SEM), and spatial representation with the support of Geographic Information System (GIS). From a large-scale (n = 400) investigation in the Moc Chau district, the statistical data extracted from the survey provides input data for the score determination process. SEM analysis shows that each landscape site has 11 determinants influencing the landscape value assessment. Using the RMSE comparison (for validation) with different interpolation methods, the ordinary kriging method is chosen to model the aggregation landscape value map of Moc Chau District. About 24.97% total area of the study area has great potential for tourism development, being mainly distributed in the center of a high mountainous area. This approach can be used as a model to advocate local and regional assessment and enhance value-based management in other territories in Vietnam and beyond.

## Introduction

*Landscape value* plays an operational role in the relationship between geographical characters and social and cultural perception of landscapes [1,2]. It highlights the importance of human society and its interaction with biophysical factors [3,4], which therefore reflects comprehensive *landscape value assessments* for efficient decision making [5]. Through harmonizing opinions from the perspective of stakeholders, landscape value assessment contributes to the process of territory planning and development [4,6,7], natural resources management [8,9], natural risk management [7], and assessments of socio-economic development [10–12]. This is a multi-dimensional approach for considering several aspects of landscape value, covering both the human and nature landscape [13]. Particularly, the approach is powerful for evaluating principal values in vulnerable areas such as biological conservation [14] and rangeland [15]. However, the diversity of landscape value causes a misidentification of crucial values, which results in conflicts in the process of landscape planning [3]. The approach raises conflicts in terms of visions, functions, and interests as a result of divergent views [16]. A wide range of social and cultural factors (e.g. education, income, and culture) can partly explain this problem, which directly and indirectly impacts the opinions of respondents [17]. Therefore, the selection of core landscape values and influencing elements support the determination of priorities in process of decision-making [4,18].

Multi-criteria analysis (MCA) is the widest applied method for quantifying landscape values [19]. However, the process of assessment depends on the personal views and experiences of decision-makers [20], which causes uncertainties and creates inaccurate assessment models [21]. Therefore, implementing SEM performs a comprehensive, objective, and flexible assessment through testing the fits of the research hypothesis and real data [15,22]. SEM is a popular statistical approach for understanding complex interactions among physical components of the natural landscape and examining multi-dimensional interactions of nature-human relationships. The approach allows relating different aspects of environmental issues through constructing model structure and path analysis. Compared to several techniques of MCA (e.g., TOPSIS and AHP), SEM generates more precise and reliable output using data derived from different sources (e.g., social surveys and statistical data). The method detected a wide range of landscape issues such as the influence of landscape patterns on the environmental quality [23], wetland restoration [24], estimating landscape values [15], the effect of climate changes on ecosystems [25], examining the degradation of ecosystems [26], assessing the human-climate effects on ecosystems [27,28]. In recent years, the integration of SEM and GIS is a novel application for potential risk assessment [29], predicting the distribution of soil [30], mapping groundwater quality [31]. With the development of geographical information systems (GIS), integrating spatial methods for modeling landscape values allows identifying and displaying the allocation of attributes explicitly [9,13]. Additionally, to identify underlying complex and abstract relationships among factors, the combination allows estimating values in unsampled points based on different methods of spatial analysis. Otherwise, the complexity of SEM and a shortage of data availability would prevent the application of SEM-GIS integration in landscape studies. However, the proposed method is probable to be further developed due to its advantageous abilities to solve complicated problems of landscape research. The techniques can show a partial or comprehensive estimation of landscape values, which then supports the preferences of management and planning [1,12]. Coupling with the participatory approach, this enables the contribution of stakeholders to the process of decision-making through assessing place-based values [2]. Therefore, it appraises the priority in landscape management from the view of human interaction on the landscape [4].

Among landscape values, potential tourism is one of the most complex values and is easy to misidentify the influencing element [13]. In Vietnam, Moc Chau is a representative area for eco-tourism development thanks to its natural landscape and cultural identity. The diversity of landscape values in both terms of nature and culture advocates the potential for tourism development in the study area. Therefore, the identification of significant landscape values for eco-tourism development supports the process of territory planning and the formation of strategies for development. Aiming at identifying potential landscape values from the perspective of stakeholders, our study integrates quantitative and spatial analyses for conducting comprehensive assessments. Based on surveying the opinions of native people, we employ structural equation modeling (SEM) for understanding significant factors and underlying dimensions of landscape characters. Following, we apply GIS to interpolate the distribution of landscape values for the entire study area. While most previous studies on landscape value assessment focused on physical aspects, our study contributes a novel approach to estimating landscape values in terms of socio-economic views. In landscape research, the integration of stakeholder survey, SEM, and GIS seem not to be applied for identifying landscape values. The proposed method allows reducing uncertainties when considering the complexity, abstraction, and multi-dimensions of social and cultural landscape value. Although existing uncertainties occur in spatial analysis, the proposed method allowed predicting the landscape value of the entire study area, which therefore suggested hot spots and cool spots for potential eco-tourism development. Despite the complexity of SEM and a shortage of data, the method is the potential to become the foundation for evolutions of more precise and accurate methods and approaches in the future. In addition to the first part (introduction), the remaining paper is organized as follows: *(ii)* The multidimensional values in an ecotourism landscape; *(iii)* Study area, data resources, and methods; *(iv)* Results; and (v) Discussion and Conclusion.

## The multidimensional values in an ecotourism landscape

### Landscape values and multidimensional approach

Landscape and its intrinsic values play a significant role in landscape planning and solving development conflicts [3]. In 1991, landscape values were first proposed by Rolston III and Coufal with 10 principal values [32] and then were recommended two additional values by Brown and Reed in 2000 [33]. Until 2006, the concept of landscape value as *“an operational bridge between the geography of place and sense of place*” was offered by Brown (6), which then motivates the identification of landscape values for territory development preferences in different fields. Following, landscape value assessment has become wider applied in the process of decision-making at various temporal and spatial scales.

Conventionally, landscape value assessment focus on a specific aspect within a disciplinary approach, which lacks convergences and interactions in research contributions [34]. Ha and Yang (2019) developed a universal system for evaluating the landscape aesthetic of Natural World Heritage Site with the integration of MCDM and GIS [35]. Riechers et al. (2020) revealed the deterioration of recreational value as a consequence of landscape simplification through an in-depth analysis of four cases, which thus suggested and supported landscape restoration and management [36]. Sharafatmandrad and Khosravi Mashizi (2020) examined the aesthetic of landscape value using SEM with data derived from stakeholder surveys to consider essential interactions for efficient environmental planning [15]. Karasov et al. (2020) measured scenic values using geolocated social media data for the digital landscape model, which allows investigating landscape coherence for landscape planning and management in the National Park Peneda-Gerês (Northern Portugal) [37]. Miller et al. (2021) clarified different provision service values of Western Mau Forest through participatory mapping and semi-structured interviews, which therefore identify significant factors for avocating resource management and conservation [38]. Most landscape assessments focusing on reflecting visual values landscape such as aesthetics and recreation. However, the individual landscape is featured by a wide range of values, in which a value either interacts or constrains others [34]. Therefore, existing conflicts appear in the process of landscape planning, which influences the identification of development priorities.

The diversity and complexity of landscape values promote the adaption of a multidimensional approach for assessment, which allows valuing landscape through harmonizing multi-disciplinary views [39]. Cerveny et al. (2017) identified landscape value typologies and mapped valuable sites of the Olympic Peninsula (Washington, USA) using social values from community meetings and PPGIS, which thus advocated strategies for planning and management in diverse and complex territories [13]. Fagerholm et al. (2017) [2] revealed the human interaction on the natural landscape for integrated management through identifying the common patterns in the opinions about landscape values obtained from GIS surveys and spatial analysis [2]. Ernoul et al. (2018) examine landscape values hotspots through bivariate heat maps of concurrent values in biodiversity hotspots of the Camargue Biosphere Reserve (southern France) using data from maptionnaire [40]. Chen et al. (2018) carried out a spatial pattern of landscape value distribution using social media data, which therefore contributed to social impact assessment of the hydroelectric dams for efficient decision making [11]. Plieninger et al. (2018) identified and mapped landscape values using open survey questions and spatial analysis for determining development priorities to solve potential land-use conflicts [4]. Generally, multidimensional value assessment examined social and cultural landscape values, which are abstract and intangible. Therefore, it advocates the role of stakeholders in valuing landscape for identifying the development preferences and/or conflicts [41].

### The determination of potential aggregation values in an ecotourism landscape

Worldwide, ecotourism development has become a principle strategy to archive sustainable development [42]. The estimation of landscape value for potential tourism development is one of the most popular tasks [6], which arises from multiple components shaping the landscape. A landscape with recreation potential encompasses not only nature and social identities [43], but also developing components such as infrastructure [44], and strategies [45]. A separate assessment of individual sections reflects partial potential development, which therefore advocates a measurement of aggregation values for sufficient and reliable assessments under specific research contexts and development demands.

Aggregation landscape value assessment integrates views and perceptions of experts and stakeholders, which thus allows conducting interdisciplinary analyses. Landscape values depend on not only physical opinions but also cultural and social insights under a variety of assessment contexts and development preferences [46]. Therefore, the multidimensional approach enables performing the changes involvement from the individual or sectorial point of view into subjective and exhaustive consideration [3]. From the socio-economic views, the identification of landscape values could be clarified through two popular methods, including surveying perceptions on the landscape, and mapping place-based values of special sites [37,47]. Value mapping methodology has first developed by Brown and Reed in 2009 [9], which integrates the two methods for effective estimation of landscape values. In this sense, landscape values depend on not only individual opinions but also cultural and social insights under a variety of assessment contexts and development preferences [46]. Aggregation landscape value assessment integrates views and perceptions of experts and stakeholders, which thus allows conducting interdisciplinary analyses. Therefore, the multidimensional approach enables performing the changes involvement from the individual or sectorial point of view into subjective and exhaustive consideration [3]. An estimation of aggregation values executes representative landscape values of stakeholder’s views, interrelating, and harmonizing various perspectives. Therefore, implementing multi-criteria decision-making (MCDM) has been widespread thanks to accurate and flexible assessment. A wide range of quantitative method (e.g. AHP [48] and SEM [15]) allows combining aspects of research objects for multi-dimensional analysis.

In recent years, with the support of GIS techniques, spatial decision-making has been further examined in the process of territory planning and identifying priorities in development strategies [6,12,49]. Integrating spatial analysis allows in-depth investigating the interrelation between social-cultural perception and place-based values, which therefore reinforces the efficiency of development strategies [10]. The approach allows considering the transformation of landscape values corresponding to the spatial landscape changes in assessment contexts, thus identifying vulnerable areas [50], hot spots, and/or cool spots in the development preferences [51]. Therefore, mapping multidimensional values suggests conflicts and/or suitability in the consideration of development, which becomes fundamental for decision-making [4,49].

## Study area, data resources, and methods

### Study area

**Fig 1:**
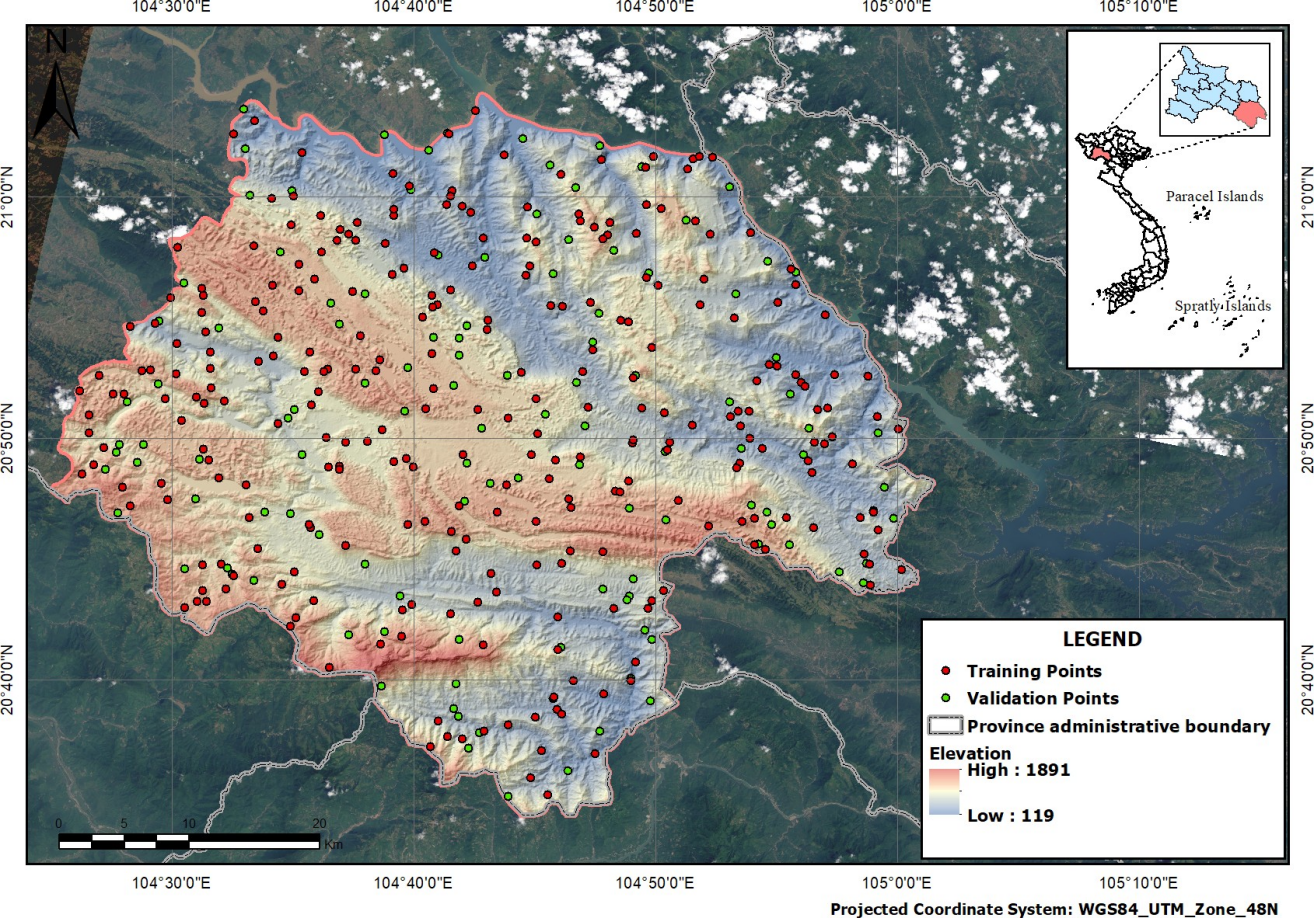
Location of study area.

Moc Chau District (20^o^63’ N and 104^o^30’– 105^o^7’E) is in the northern mountainous region of Son La (Vietnam) (as shown in Fig 1). This area is featured by a wide range of hills, mountains, plateaus, and pan-shape valleys combining with a complex hydrologic system. The diversification of topography brings about a remarkable and stunning landscape for tourism attraction. It is sited at 1050m high above sea level and is characterized as a sub-temperate climate with pleasant weather. Additionally, this is settlement areas of different ethnic groups in Vietnam such as Thai, H’Mong, and Muong, which therefore brings about cultural diversity with a variety of traditional identities. Therefore, Moc Chau has great potential for developing eco-tourism in both terms of physical and cultural landscapes. The assessment of tourism potential is a significant task for identifying development priorities and preferences in the process of landscape planning and management.

### Data resources

Our data resources are derived primarily from stakeholder interviews conducted from March to May 2020. This comprises three components: *(i)* Socio-demographic information of interviews. *(ii)* Landscape assessments from the perspective of stakeholders with a 7-points Likert scale (from none to the highest value). It includes 25 questions about various landscape values and collects responses from native people. *(iii)* The spatial data referring to the distribution of observed landscape sites are acquired by GPS when interviewing local people. Besides, other spatial data used in this study, including administration data (download at Diva-GIS website: https://www.diva-gis.org/Data) and Digital Elevation Model and Satellite image (download at USGS Earth Explorer website: https://earthexplorer.usgs.gov), are free and public.

### Methods

**Fig 2:**
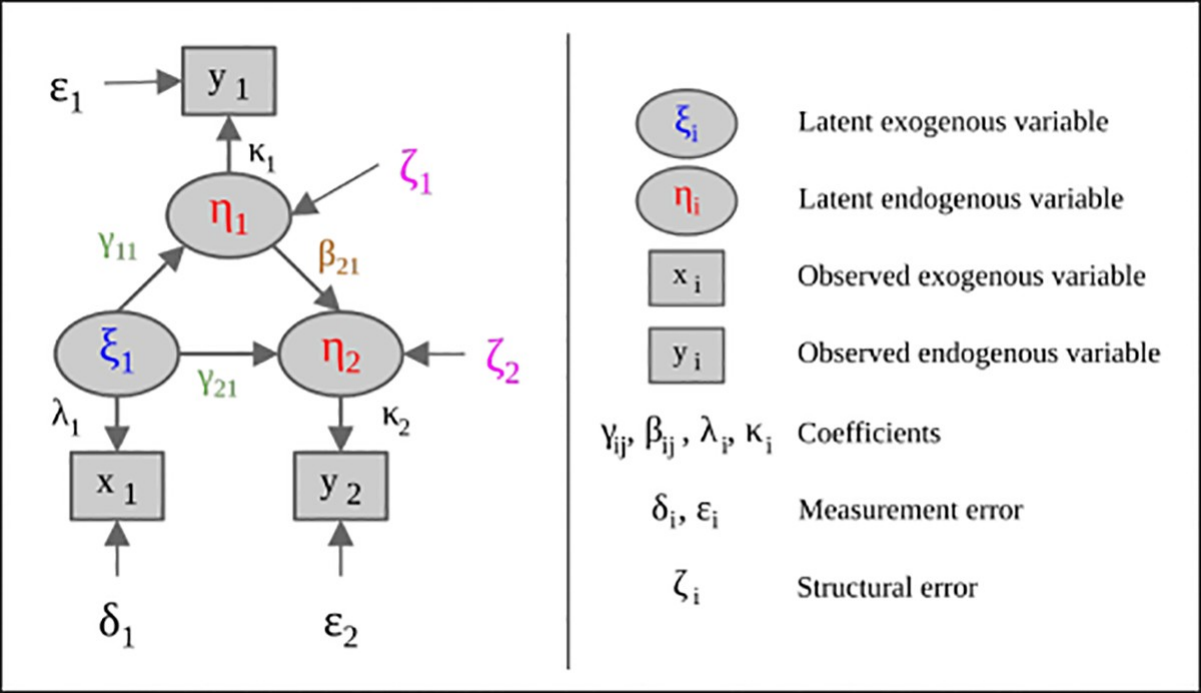
The study flowchart employed in this study.

The proposed method can be summarized as follows: *(i)* the conduction of a stakeholder survey that collects landscape value assessments from native people, *(ii)* An analysis of data by SEM approach, *(iii)* Mapping the potential aggregation values with GIS. As shown in Fig 2, the proposed method performs the aggregation values of ecotourism landscape, consisting of the following three steps:

#### Step 1: Model design and data collection

This study determined the primary research objects, which then developed a conceptual model and selected variable indicators for investigating the opinions and views on landscape values of the study area. Before conducting field interviews, it designed the stakeholder surveys and conducted sample selection. The stakeholder survey is recorded by written materials through either face-to-face interviews or teleperformance interviews (in remote landscape sites), which then was transformed into IBM SPSS Statistic 23 for further analysis.

#### Step 2: An analysis of data by SEM approach

This study conducted pre-processing to eliminate surveys with missing data, displaying spatial sampling, and carried out demography sample description. Following, it performed reliability tests by Cronbach’s alpha before employing Exploratory Analysis and Confirmation Factor Analysis. After that, it estimated aggregated values of eco-tourism landscape by SEM, which then were validated by different goodness-fit indices and the model error. If these indices do not meet the requirement for a best-fit model, the model will be performed again.

#### Step 3: Mapping the potential aggregation values with GIS

Based on the output of the SEM model, this study calculates the aggregated value of individual landscape sites. Then, it uses 70% of point-sample for interpolating landscape values in GIS environmental through a wide range of interpolation techniques. Following, it validates the model by comparing interpolated values and calculated values from a 30%-point sample. At this stage, it compares the modeling error indices from different interpolation techniques, which therefore allows selecting the suitable one for mapping landscape values.

#### Stakeholder survey methods used in this study

In this study, primary data were acquired from a stakeholder survey, which was developed based on the landscape value assessment offered by Raymond and Brown [52]. To reflect the landscape value, several influencing elements were selected based on a literature review on eco-tourism. Therefore, the elements were indicators for describing abstracts meaning of landscape values to avoid incorrect identification from native people.

Except for questions related to socio-demographical information (including name, gender, age, working status), four selected values (Tourism, Aesthetic, Economics, and Cultural Heritage) were clarified based on 25 questions corresponding to influencing elements of eco-tourism development. Following, an overall assessment of landscape value from respondents (Q26) was conducted to summarize their general opinions. The questions used a 7-point Likert scale, with values ranging from very low impact to very high impact for quantifying the landscape values. Table 1 showed adequate questions for collecting landscape assessment from stakeholder surveys. The investigation results from stakeholder surveys are detailed in S2 File.

**Table 1:** Describing landscape values for tourism used in the stakeholder surveys for landscape assessment.

Landscape value	Influencing indicator	
**Aesthetic/Scenic**	(Q1) Breathtaking scenery and natural attractions [1, 6]	(Q4) Actions for promoting nature conservation [5]
	(Q2) Pleasant weather [1]	(Q5) Clean and tidy environment [1]
	(Q3) Scenic mountain and valleys [1]	
**Economics**	(Q6) Ecotourism activities for constructing environmental awareness and respect [5]	(Q8) Wide selection of restaurants/cuisine [1]
	(Q7) Wide choice of accommodations [1]	(Q9) Wide variety of shop facilities [1]
**Cultural Heritage**	(Q10) Distinctive history and heritage [1, 6]	(Q14) Traditional handicraft [3, 6]
	(Q11) Variety of special events/festivals [1]	(Q15) Indigenous knowledge [3]
	(Q13) Beautiful costumes and architectures [1,6]	(Q16) Local tourism products [3, 2]
**Recreation/Tourism**	(Q16) Diversity of tourism sites [1]	(Q31) Availability of travel information [1, 4]
	(Q17) Friendliness of service [1]	(Q33) Convenient accessibility [1,5]
	(Q18) Friendly and helpful local people [1]	(Q34) Helpfulness of welcome center [1, 4]
	(Q19) Local tour guides [4, 5]	(Q35) Convenience of local transportation [1]
	(Q30) Local participants in tourism activities [4, 5]	(Q36) Well communicated traffic flow [1]
**Overall Assessment (Q26)**		

**References:** [1] **Chi and Qu [53]**, [2] **Cassatella [54]**, [3] **Yang, Ryan [55]**, [4] **Sonchaem, Phuditthanawong [56]**, [5] **Tseng, Lin [57]**, [6] **Kalaycı Önaç and Birişçi [58]**.

In this study, we conducted two processes of sampling selection, including demographic and spatial ones. The target respondents were native people in the study area. At the first stage of sampling selection, for sample size determination, this study employed the confidence interval approach, which has been widely applied in social research. The equation below shows the calculation of sample size with 95% accuracy at a 95% confidence level:

$$n=\frac{z^{2}(pq)}{e^{2}}=\frac{1.96^{2}(0.5x0.5)}{0.05^{2}}=385$$ (1)

*Where*: z figures the standard error corresponding to confidence level (95%), p is the variability in the population (50%); q = 1-p; and e indicates the allowable error (±5%). This study supposed that there would be only a 95% response rate; and about 5% of the total survey result would be inappropriate. Therefore, a total of 427 people is selected to answer the survey.

For the spatial sampling selection, this study randomly chose 427 observation points representing to tentative positions of respondents for asking about landscape value assessment surrounding them. The fixed coordinate values of observation points were collected by GPS, which depended on the actual locations of surveying in the field trip. During field trips (from 30^th^ March to 30^th^ May 2020), inaccessible points were eliminated from the spatial sampling due to the complex topography. To clarify the accurate and precise landscape values for potential tourism development, our study recruited participants who have studied and worked in the tourism sector. After removing surveys with missing data, there were 400 respondents for assessing aggregate landscape values in the study areas (the socio-demographic information is shown in Table 2). In the Moc Chau district, approximately 5000 people have studied and worked in the tourism sector in 2019, including tour guides, owners and staffs of service supplies in tourism (e.g. restaurants, hotels, homestays, and motels), and officers of tourism sites. Our study selected 400 participants to take part in the stakeholder survey, accounting for about 8% of people working in the tourism sector. They understand nature and human landscape values as well as current tourism activities in the study areas. Additionally, they are influenced strongly by changes in tourism development and investment in Moc Chau District. The process of participant recruitment for participant recruitment is supported and under control by the Moc Chau National Tourism Management Board and local authorities to ensure the quality of data collection from stakeholder surveys. Therefore, their perceptions and insights contribute significant views and reliable assessments to the identification of landscape values, which makes the recruited sampling become representative of a larger population.

**Table 2:** Socio-demographic statistical descriptors of the respondents (n = 400).

Variable	Category	n	%	Mean	Std. Deviation
**Gender**	Female	166	41.5		
	Male	234	58.5		
**Age**				28.311	9.864
**Working status**	Studying	37	9.25		
	Working	325	81.25		
	Unemployment	16	4.0		
	Retired	22	5.5		

With a specific research object, our study improves the understanding of landscape science on determining landscape values existing in geographical territories. However, dilemmas are existing in human perceptions partly because goals, the rights, and the welfare of individuals influence personal opinions. Despite the limitation, the opinions of participants show the residents’ understanding of the intrinsic values of the landscape. According to The Belmont Report about the Protection of Human Subjects of Biomedical and Behavioral Research issued Ethical Principles and Guidelines for the Protection of Human Subjects of Research (1979), the stakeholder survey does not need approval by an ethics committee for the following reasons: *(i)* The spatial object points were surveyed randomly to avoid potential influences of participants’ decisions. *(ii)* As mentioned in the terms of agreements before conducting the surveys, the outcomes of investigations contribute to scientific research. Thus, individual considerations and choices are preserved and do not have any risks. *(iii)* The scale of the questionnaire is from 1 to 7 (no value “0”) and does not mention monetary value, which ensures maximize possible benefits and minimize possible harms to the participants; (iv) The outcome of surveys is only employed for modeling purposes to perform a comprehensive assessment, not for conducting more/less comparations. Additionally, the advantages and disadvantages of research objects offered by individuals are significant equally thanks to random sampling.

The informed consent process provides essential ethic information to potential participants and empowers them to make a rational decision about participation. The complexity of consent documents could prevent participants from the process of gathering information. Additionally, our study examines the opinions of native people (includes those from minority ethnic communities) in the mountainous region. Some people denied participation because they could be unable to understand Vietnamese, or could lack free time, or could not interested in the research issues. Therefore, the agreement of participants was given verbal consent thanks to its simplicity and ease of understanding for the study participants. Meanwhile, most questions do not mention information related to their personal life. If all four criteria of information disclosure, competence, comprehension, and voluntariness aren’t satisfied, the information will be denied immediately by native people. The competence or capacity of an individual to make a decision depends on his/her ability to understand relevant information, on appreciating the nature of a situation along with its consequence, on the reason the given information, and on the ability to communicate their choice [59]. Therefore, investigators of our study are native people to ensure a “meaning” informed consent at a local scale.

#### Data analysis by SEM approach

**Exploratory Factor Analysis (EFA)** is one of the widest applied statistical tools for exploring mathematical relationships among observed variables. The method allows examining patterns of observed variables for explaining principal dimensions of research objects through linear functions (as shown in Eq 1). In this study, we employed IBM SPSS Statistics 23 for conducting EFA to detect underlying factors of aggregated landscape value assessment derived from the stakeholder survey. The method of extraction and rotation were Principal Axis Factoring and Promax Rotation with Kaiser Normalization, respectively.


$$x_{i}=\sum_{i=1}^{k}\alpha_{ij}f_{j}$$ (2)

*Where*: *x*_*i*_ are observed variables; *f*_*j*_ are common factors; *α*_*ij*_ are factor loadings, *k* is the number of factors *(k<j)*. To estimate the intercorrelation, several measurements have been applied for testing and modifying the results of EFA. Firstly, factor loadings are regression coefficients that indicate how *x*_*i*_ is interpreted by the *f*_*j*_ (being required to be more than 0.3). Secondly, the Kaiser-Meyer-Olkin (KMO) estimates the measure of sample adequacy for EFA, in which the value of KMO is required to range from 0.5 to 1. Finally, Bartlett’s Test of Sphericity considers the correlation among the entire selected variable, which is obliged to be significant at the confidence level of 95%) [60].

**Confirmatory Factor Analysis (CFA)** is the technique for examining the appropriateness of the research hypothesis and reality. Rather than statistical results, the method enables testing the consistency of measurement theory through examining the model fits. In this study, CFA performs IBM SPSS Amos 20 to test the relationship among underlying factors of aggregated landscape value derived from the results of EFA, in which we assumed extracted factors to be latent constructs. Parameters were conducted to validate the reliability and convergence of the model, encompassing significance level (t-value), internal consistency (Cronbach alpha), construct reliability (CR), and average variance extracted (AVE).


$$CR=\frac{{\left(\sum_{i=1}^{k}\lambda_{i}\right)}^{2}}{{\left(\sum_{i=1}^{k}\lambda_{i}\right)}^{2}+\sum_{i=1}^{k}\left(1-{\lambda_{i}}^{2}\right)}$$ (3)

$$AVE=\frac{\sum_{i=1}^{k}{\lambda_{i}}^{2}}{\sum_{i=1}^{k}{\lambda_{i}}^{2}+\sum_{i=1}^{k}\left(1-{\lambda_{i}}^{2}\right)}$$ (4)

*Where*: *λ*_*i*_ are the standardized regression weight *x*_*i*_, 1−*λ*_*i*_^2^ are variance of *x*_*i*_, *p* is the number of factors. To meet the construct validity, an individual construct is required to have a t-value being less than 0.05 (being significant at 95% of confidence level), and the values of Cronbach alpha being more than 0.6. Additionally, the values of both CR and AVE of individual latent constructs are required to be more than 0.5 [60].

**Fig 3:**
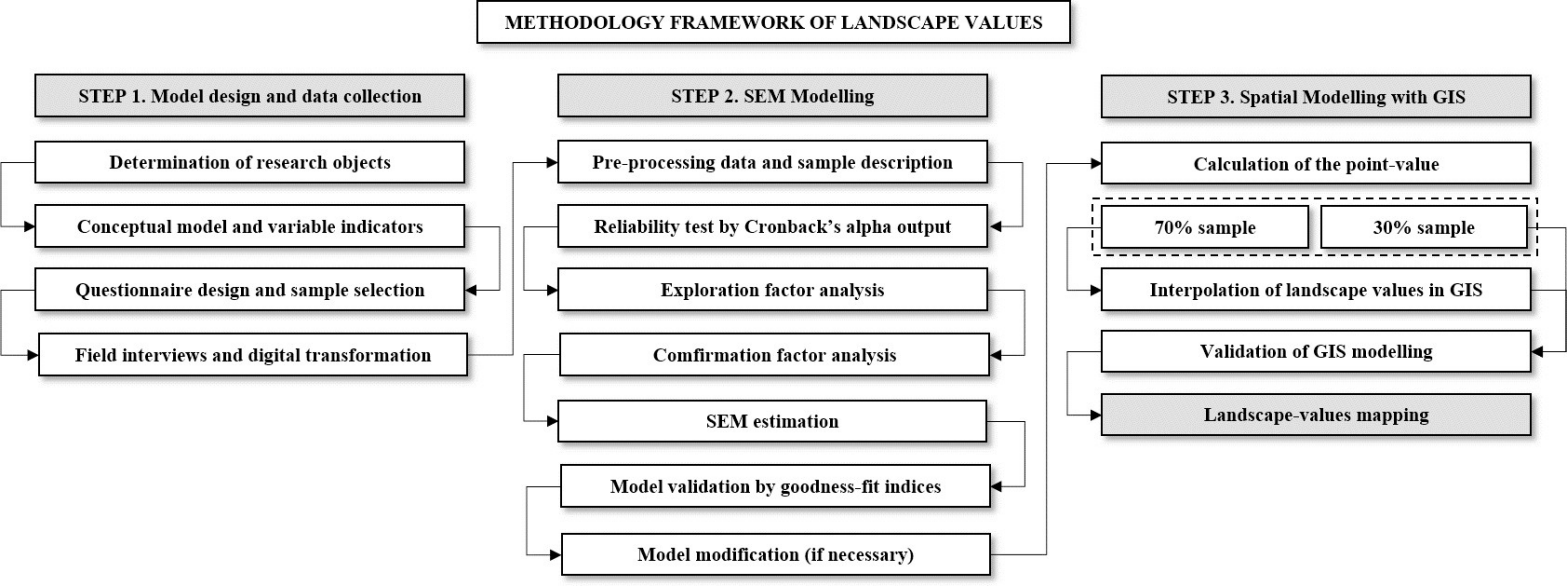
A graphical example of a structural equation model (adapted from *Joreskog and Sorbom [61]*).

**Structure equation modeling (SEM)** is a multivariate analysis method for identifying the best-fit models, which explains actual effect-casual associations based on sample data. In addition to testing research hypothesizes, the method can predict based on regression techniques [30]. To develop a structural equation model, we can illustrate a SEM producer through a graphical model (as shown in Fig 3).

The measurement model can be estimated as follow:

y=Kη+ε (5)

x=λξ+δ (6)

In this study, we use IBM SPSS Amos 20 to conduct SEM for estimating aggregated landscape values based on underlying dimensions of landscape value assessment explored from EFA and tested by CFA. The path analysis of formative variables enables predicting the aggregated value based on the result of the measurement model. Before conducting regression and prediction, our study employs several parameters for testing model fit. Firstly, the ratio of χ2 to the degree of freedom (df) of the model is required to be 5:1 or less for performing better-fitting models. The second is Goodness-of-fit (GFI) for measuring the correlation of variance and covariance in the covariance matrix. Normed fit index (NFI), Tucker Lewis Index (TLI), and Comparative Fit Index (CFI) are parameters for estimating the deviation of χ2 value between the fitted and null model. The four values are suggested to be over 0.9 to perform a best-fit model. Finally, the estimation of Root Mean Squared Error (RMSE) is employed for detecting the errors of modeling performance (Eq 7). The value of RMSE is suggested to be less than 0.1 for a fit model [60].


$$RMSE=\sqrt{\frac{\sum_{i=1}^{n}{\left({ALV}_{T}-{ALV}_{v}\right)}^{2}}{n}}$$ (7)

*Where*: *ALV*_*T*_ is the aggregation landscape value performed by interpolation; *ALV*_*T*_ is the aggregation landscape value estimated by SEM; *i* is the number of intersection units, whose number of units is n.

#### Mapping the potential aggregation values with GIS

Spatial interpolation is a popular method for estimating and compassing occurrences, allocation, orientations, and development of the physical and socio-economic phenomenon. Integrating spatial data has been wider applied for examining different environmental issues at a wide range of scales. It allows presenting continuous attributes of research objects in the study area, which can therefore forecast values at the unsampled position based on either initial points or line patterns. Our study used ArcGIS 10.4.1 for performing spatial interpolation to predict and map the aggregation landscape values based on the regression results derived from SEM. Our study applied four methods of spatial interpolation, including Inverse distance weighted (IDW), Kriging, Natural Neighbor, and Spline.

The spatial data would be divided into two categories, including the training group and validate one. The former group comprised 70% of total observed points, which then is conducted spatial interpolation for predicting aggregated landscape values through given values assessed from the perspective of stakeholders. Meanwhile, the remaining points were used for validating the results of predicting aggregation values through measuring the differences between a pair of predicted observed values and RMSE (as shown in Eq 7).

## Results

### Descriptive statistics of the stakeholder survey

Landscape values were determined according to the value of the mean. Table 3 shows absolute estimations of landscape value, which indicates the means, standard deviation, and Skewness derived from individual influencing factors. Generally, landscape values are estimated to between moderate-high and high. Q24 has the highest mean score (5.74) and most consistency agreement (lowest standard deviation value). The lowest landscape value belongs to Q15 (5.26). For the overall assessment, the landscape values of Moc Chau are 5.53 with the lowest standard deviation being 0.904.

**Table 3:** Descriptive statistics of stakeholder survey.

Landscape value	Question	Min-Max	Mean	Std. Deviation	Skewness	Question	Min-Max	Mean	Std. Deviation	Skewness
**Aesthetic/Scenic**	**1**	1–7	5.63	1.380	-1.300	**4**	1–7	5.28	1.582	-0.823
	**2**	1–7	5.63	1.391	-1.151	**5**	1–7	5.56	1.564	-1.073
	**3**	1–7	5.30	1.477	-0.720					
**Economics**	**6**	1–7	5.44	1.491	-0.878	**8**	1–7	5.53	1.365	-0.954
	**7**	1–7	5.48	1.385	-0.990	**9**	1–7	5.29	1.455	-0.809
**Cultural Heritage**	**10**	1–7	5.32	1.441	-0.951	**13**	1–7	5.65	1.352	-1.113
	**11**	1–7	5.45	1.476	-0.967	**14**	1–7	5.32	1.415	-0.997
	**12**	1–7	5.67	1.362	-1.061	**15**	1–7	5.26	1.424	-0.832
**Recreation/Tourism**	**16**	1–7	5.57	1.315	-0.977	**21**	1–7	5.58	1.417	-1.061
	**17**	1–7	5.62	1.307	-0.939	**22**	1–7	5.63	1.363	-1.117
	**18**	1–7	5.45	1.399	-0.700	**23**	1–7	5.71	1.365	-1.078
	**19**	1–7	5.58	1.359	-0.994	**24**	1–7	5.74	1.306	-1.218
	**20**	1–7	5.64	1.395	-1.071	**25**	1–7	5.48	1.418	-1.055
**Overall Assessment**	**26**	1–7	5.53	0.904	-0.474					

Our study examines the data distribution through standard deviation and Skewness for considering the consensus of stakeholders’ viewpoints. The standard deviation shows the dispersion of data, ranging from 0.904 to 1.582. Otherwise, the negative Skewness occurs in 26 questions, being between -1.300 and -0.474. According to the rule of thumb, 11 out of 26 questions show a significant divergence in opinions of landscape value for potential development with the value of Skewness being less than -1.000. The Skewness being less than -0.5 shows that the potential for eco-tourism of individual landscape sites is different, which therefore results in hot spots and cool spots in recreation. Only overall assessment (Q26) witnesses the most consistent agreement with the Skewness value being -0.474. The identification of landscape sites with great potential will advocate efficient landscape planning and territory development.

### Underlying factors for assessing landscape values

EFA extracted 16 out of 25 influencing items for assessing potential landscape values, which then were grouped into five underlying factors. Table 4 gave information on selected items and their correlations to loaded factors. Following, CFA tested the formative relationship between influencing elements of tourism potential development (items) and latent variables of aggregation landscape value (constructs). Table 5 showed the standardized regression weights of individual items (being loaded at the p<0.001 level), which then was executed reliability (Cronbach α and CR), and validity (AVE) of each construct.

**Table 4:** Underlying dimensions of landscape value assessment using principal axis factoring extraction method and Promax Rotation with Kaiser Normalization (KMO = 0.855, the significance level of Bartlett’s Test of Sphericity is 0.000, total variance explained is 53.725%).

Factor	Items	Factor Loadings	Factor	Items	Factor Loadings
**Tourism**	Q19	0.814	**Culture**	Q14	0.835
	Q20	0.726		Q15	0.748
	Q21	0.678		Q13	0.711
	Q18	0.583	**Accessibility**	Q25	0.802
**Strategies**	Q4	0.801		Q24	0.649
	Q8	0.610			
	Q6	0.558	**Nature Landscape**	Q2	0.745
	Q9	0.544		Q1	0.621
	Q5	0.500			

**Table 5:** Reliability test and convergent validity of the measurement model.

Constructs	Items	Standardized Regression Weight	Reliability		Validity
			α	CR	AVE
**Tourism**	Q19	0.771	**0.817**	**0.755**	**0.513**
	Q20	0.690			
	Q21	0.674			
	Q18	0.725			
**Strategies**	Q4	0.547	**0.759**	**0.763**	**0.396**
	Q8	0.684			
	Q6	0.699			
	Q9	0.700			
	Q5	0.485			
**Culture**	Q14	0.813	**0.821**	**0.832**	**0.607**
	Q15	0.757			
	Q13	0.767			
**Accessibility**	Q24	0.813	**0.749**	**0.752**	**0.603**
	Q25	0.738			
**Nature Landscape**	Q2	0.666	**0.669**	**0.672**	**0.508**
	Q1	0.756			

Based on the result of EFA, 4 out of 16 extracted items loaded onto the latent variable for tourism. The factor reflected the provision of tourism services, in which three variables related to the participation of local people in tourism developed to have the highest value of factor loading (Q19, Q20, and Q18). The standardized regression weights estimated in CFA ranged from 0.690 to 0.771, whereas Cronbach α, CR, and AVE met the requirement for reliability test and convergence validation.

The latent variable for strategies loaded four variables, describing actions for ecotourism development in terms of environment protection, and tourism service improvement. Through CFA, the measured variables loaded onto the factor loading being between 0.485 and 0.7. Despite the strong reliability, the validity showed a divergence (the AVE value is less than 0.5), which then was excluded from the measurement model.

EFA found three items for assessing the factor of culture, which emphasized the transformation of traditional and cultural values into tourism products (Q14, Q15, and Q13). The factor of accessibility included two variables (Q24 and Q25), which reflected the promotion of transportation services for better approachability to tourism sites. Two variables were used to develop the factor of natural landscape central in attractions of scenery and weather in the study (Q2 and Q3). According to the results of CFA, items that were used to develop latent variables for culture, accessibility, and natural landscape were loaded with the standardized regression weights being from 0.666 to 0.813. Additionally, the values of Cronbach α, CR, and AVE estimated from the four constructs show consistency reliability and convergence validity.

**Fig 4:**
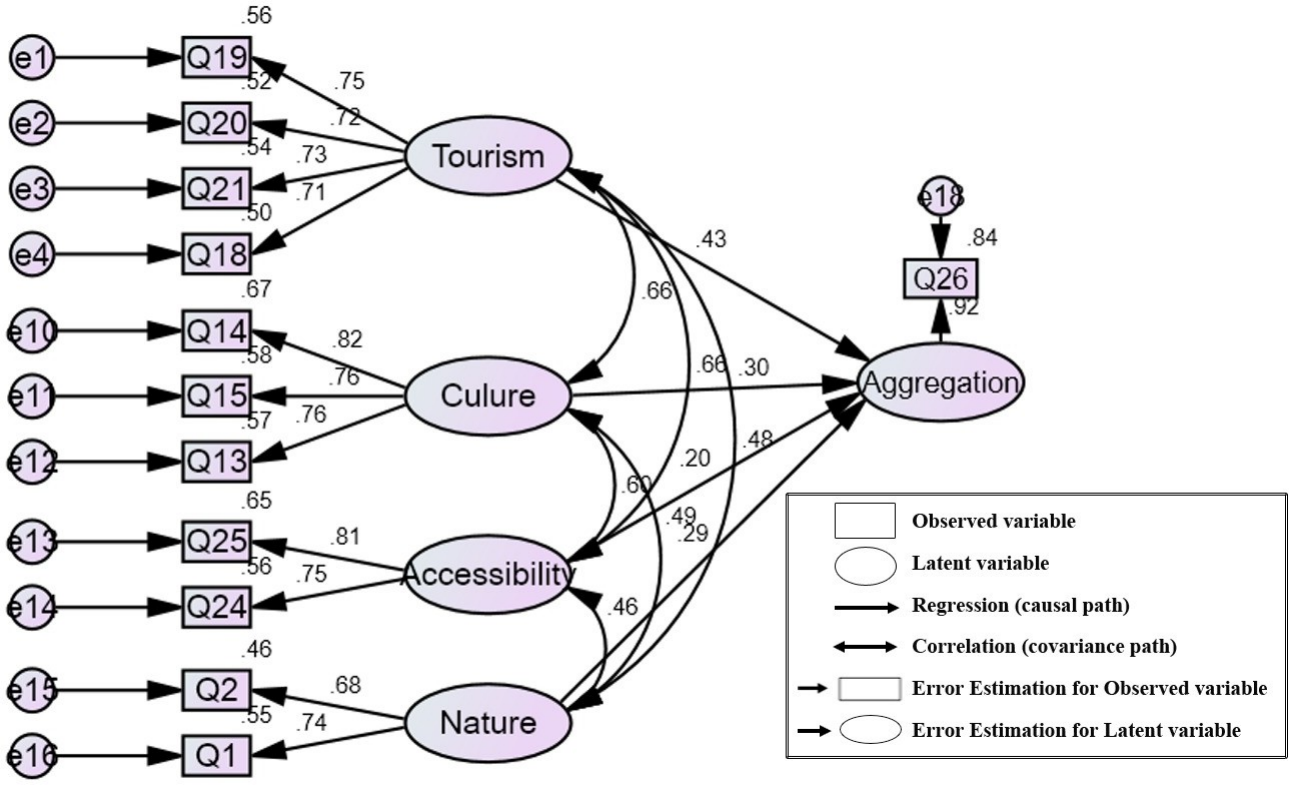
Structural model parameters for modeling the overall assessment of aggregation landscape values (χ^2^/df = 3.381, GFI = 0.927, TLI = 0.908, CFI = 0.934, RMSE = 0.084).

The structural model for modeling the overall assessment of aggregation landscape values is showed in Fig 4. Four indices for testing model fit (χ2/df, GFI, TLI, CFI) and the model error (RMSE) met the requirement for a best-fit model. The standardized regression weights were estimated at the p-value<0.001, figuring out what extends individual construct, and each item influences estimations of aggregation values. Tourism affected most of the aggregation values (0.43), following by Culture (0.30), Nature (0.29), and Accessibility (0.2). These standardized regression weights illustrated positive effects on the aggregation values, explaining about 92% of the aggregated values.

### Mapping aggregation landscape values for eco-tourism development

The selection and validation of the interpolation method are primarily for predicting aggregated landscape values. Our study used RMSE for comparing model errors among spatial estimations derived from different interpolation techniques. 118 out of 400 points were selected randomly for conducting the deviation between observed and predicted values (as shown in Table 6). The ordinary Kriging technique with the Spherical semivariogram model performed the lowest value of RMSE, which became the most successful method for interpolating the landscape values. While the observed aggregated values performed by SEM results ranged from 2.708 to 6.720, the predicted values derived through the interpolation algorithm fluctuated between 4.711 and 5.873. However, the mean and sum of the 118 values estimated in both training and validation groups are approximate, with the former being 5.288 and 623.964, and the latter equaling 5.295 and 624.831.

**Table 6:** An estimation of RMSE corresponding to interpolation methods.

Interpolation method		RMSE
**IDW**		1.085
**Ordinary Kriging**	Spherical semivariogram model	1.009
	Circular semivariogram model	1.010
	Exponential semivariogram model	1.022
	Gaussian semivariogram model	1.029
	Linear semivariogram model	1.013
**Nature Neighborhood**		1.123
**Spline**		1.380

**Fig 5:**
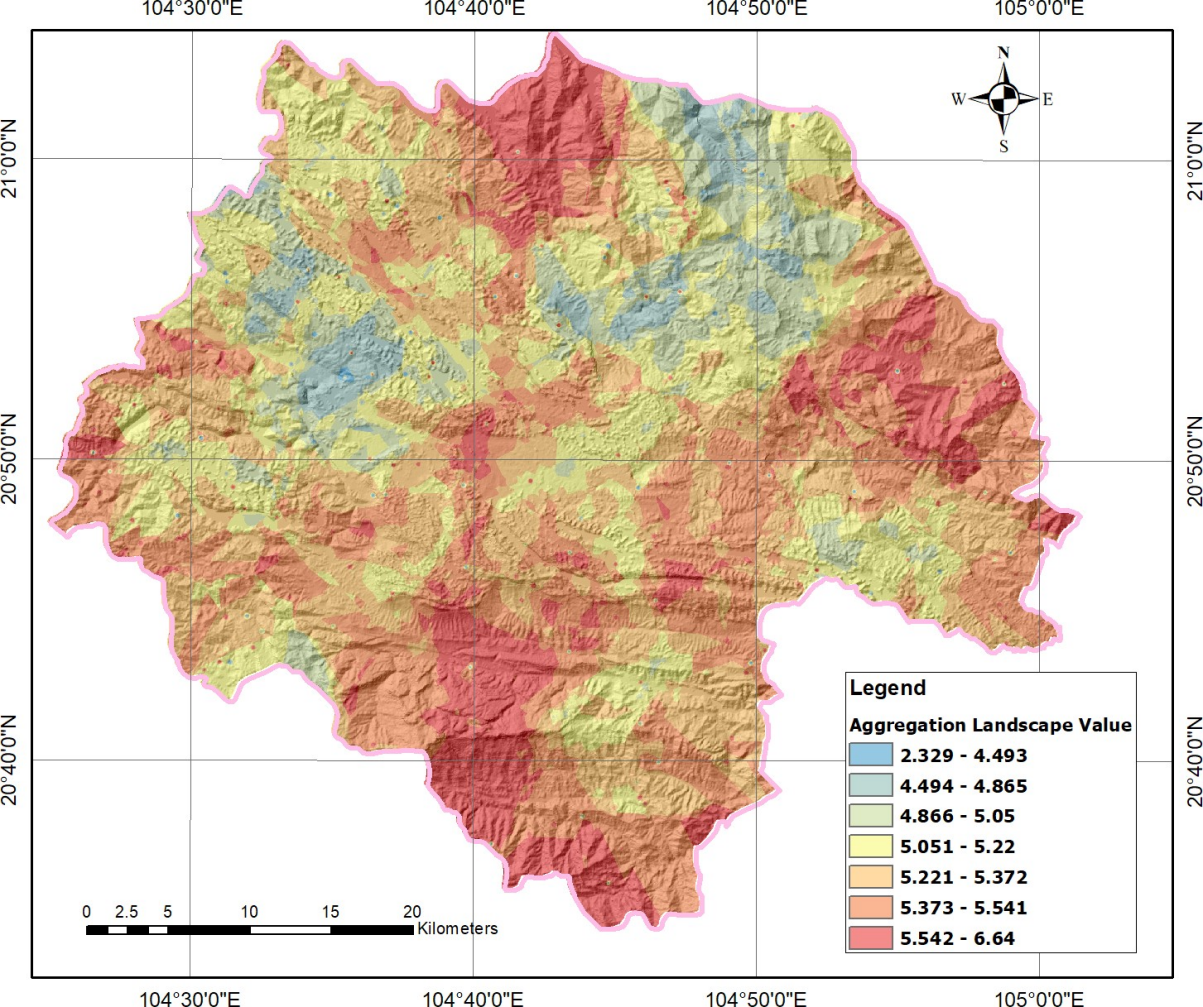
Aggregation landscape value map of Moc Chau District (using ordinary kriging method of interpolation with the spherical semivariogram model).

Based on the ordinary kriging method of Interpolation with the spherical semivariogram model, the aggregation landscape value map of the Moc Chau District was illustrated (Fig 5). The aggregated values ranged from relatively low (2.329) to extremely high (6.64). On average, the relatively high mean value (5.299) showed great potential for eco-tourism development, whereas the low standard deviation value (0.225) indicated homologous conditions for tourism investments in Moc Chau District.

The tourism landscape value in the North, East, and South of Moc Chau is from high to extremely high aggregated values for potential ecotourism. The pan-shape valley of central Moc Chau is the residence of Thai ethnic people, which is the reason for a considerable number of cultural tourism sites in this area such as Thai traditional houses, festivals, and cuisine. Additionally, the valley is where a majority of tourism services such as hotels and restaurants are distributed because of relatively flat topography. Thanks to being close to National Route 6 and National Route 43, ease of access is another advantage for tourism development.

High mountains in the center of the study area are between moderate and relatively high (from 4.866 to 5.372). Despite high topography, there are several attractive and aesthetic natural landscapes, which is the reason for the occurrence of tourism sites in the mountains. Besides, the mountains and hills are habitations of many ethnic groups, which brings about special and diversified cultural landscape values. The great potential for tourism development has promoted infrastructure investments for improving accessibility to attractive tourism sites in Moc Chau District. In contrast, areas having low to moderate aggregated landscape value (from between 2.329 and 4.865) distributed fragmentally in the center and North East of the study area, along high mountains and hills.

## Conclusion and discussion

### Conclusion

Our study presents an approach that integrates stakeholder survey, SEM, and GIS for predicting aggregated landscape values. A case study of Moc Chau shows its appropriateness for assessing ecotourism potentials, which advocates development preferences in this area. With the support of SEM, our study developed a theoretical framework for estimating aggregated values based on actual data, which then operated the values under a specific assessment context. Integrating GIS enables identifying priorities areas for development, which optimized the landscape values and existing infrastructure for tourism investment.

### Discussion

To highlight the interdisciplinary approach, our study employed stakeholder surveys for the multidimensional assessment of landscape values for potential tourism development in Moc Chau District. The approach is customized and changeable, depending on research goals and the specific context of assessment. Through the sampling technique, results from the stakeholder surveys integrated opinions and perceptions of decision-makers, which then would transform from individual to sectorial or social views. Therefore, the landscape value assessment would be more comprehensive and subjective, which provides actual data for subsequent analyses. Although this approach is easy to use for collecting views and identifying perceptions from decision-makers, their assessment could be inaccurate, imprecise, and subjective partly due to misunderstanding in the meaning of influencing elements. To deal with the uncertainty of stakeholder surveys, several techniques that allow evaluating expert’s opinions are suggested to be applied for performing reliable data such as the Delphi technique [58]. Additionally, the integration of physical and social variables is recommended to improve the comprehension of landscape value prediction. Besides, the aggregated value of landscape ranges from 1 to 7 because our study collected all landscape values based on a 7-point Likert scale. Therefore, a further investigation of the limitation of individual landscape value is recommended for improving the final results.

In this study, the SEM technique identifies the intercorrelations among landscape values, which allows forecasting changes in the aggregated values under different interventions of decision-making. As SEM is occasionally applied for prediction, existing limitations occur in the study flow. Firstly, the estimation derived from SEM tends to neglect errors of the measurement model, which might lead to either overestimate or underestimate landscape values in many points. Additionally, there should be an estimation of the coefficient among latent variables for testing the multicollinearity in the prediction model. Secondly, our study implied the technique for estimating aggregated landscape value from multidimensional value assessments gained from stakeholder surveys. The structure model was build based on actual data, showing interactions of different values, functions, and influencing elements of the landscape under specific assessment context and development preference. However, the technique does not reflect the influences of spatial correlation, which therefore is recommended to integrate spatial analysis for mapping aggregated values. Finally, the correlation among variables and their contributions to the aggregated landscape value is primarily in the whole study flow, in which slight changes in different stages of adopted methods conducted different results. In particular, instead of choosing the principal axis factoring extraction method with Promax rotation, principal component analysis with Varimax rotation could be selected for identifying the underlying factor of landscape value assessments. Approaches to removing variables for EFA and purifying constructs for CFA impact significantly on the estimation of aggregated value. Additionally, the employment of Monte-Carlo could be employed for changing the number of iterations in the process of EFA, CFA, and SEM for evaluating the accuracy and precision of performed results.

With the development of GIS, interpolation methods have been wider applied for conducting an overall assessment of the whole study area. Therefore, instead of comparing attributes and values of observed points, the technique enables collating potential among zones and understanding effects derived from physical, social, and cultural factors on the estimated aggregated values. Our study assessed the efficiency of predicting landscape values from eight methods of interpolations through the value of model errors. This allows large-scale estimation of aggregated values and then mapping and visualizing differences of values among zones.

## Supporting information

S1 File The questionnaire used in this study. Please find our attachment (StakeholderSurvey.pdf) for full-text stakeholder survey in both Vietnamese and English.
(DOCX)

S2 File The investigation results from stakeholder surveys of landscape value assessment for potential ecotourism development in Moc Chau district, Vietnam. Please find our attachment (SL_MC.sav) for adequate investigation results.
(RAR)

S3 File Mapping data for Fig 1. Fig 1: Administration data is used from Diva-GIS project (public domain) https://www.diva-gis.org/Data; Digital Elevation Model and Satellite image (Landsat 8) are used from USGS Earth Explorer (public domain) https://earthexplorer.usgs.gov; and point data is established by the authors.
(RAR)

S4 File Mapping data for Fig 5. Fig 5: Administration data is used from Diva-GIS project (public domain) https://www.diva-gis.org/Data; Digital Elevation Model is used from USGS Earth Explorer (public domain) https://earthexplorer.usgs.gov.
(RAR)
