# Supplementary material for: Mapping the potential aggregation values of ecotourism landscapes from stakeholder survey, structural equation modeling and GIS: Case study of Moc Chau site, Vietnam
Source: PLoS One. 2021 Jul 1;16(7):e0253908. doi: 10.1371/journal.pone.0253908 (PMC8248734; doi:10.1371/journal.pone.0253908)
Supplement: S1 File — Please find our attachment (StakeholderSurvey.pdf) for full-text stakeholder survey in both Vietnamese and English. (DOCX) [file pone.0253908.s001.docx]

PHIẾU KHẢO SÁT

**ĐÁNH GIÁ TIỀM NĂNG PHÁT TRIỂN DU LỊCH SINH THÁI**

**TẠI HUYỆN MỘC CHÂU, TỈNH SƠN LA**

**Xin kính chào Anh/ chị!**

Với mong muốn khảo sát các tiềm năng cua Cao nguyên Mộc Châu, anh/chị vui lòng cho biết ý kiến của anh/chị về các phát biểu dưới đây. Những thông tin mà Anh/ chị cung cấp phục vụ cho mục đích điều tra, đánh giá, trong đề tài khoa học của sinh viên Trường Đại học Tây Bắc và công bố các bài báo khoa học phục vụ phát triển du lịch của địa phương, ngoài ra không vì một mục đích nào khác.

**Anh/chị vui lòng cho biết một số thông tin cá nhân sau:**

*Họ và tên:* _____________________________________________ *Tuổi:* ______ *Giới tính:* 🞏 Nam 🞏 Nữ

Nghề nghiệp: 🞏 Sinh viên/Thực tập sinh ngành du lịch
🞏 Đang làm việc tại các đơn vị cung cấp dịch vụ du lịch
🞏 Tạm nghỉ việc hoặc nhân viên thời vụ tại các đơn vị cung cấp dịch vụ du lịch
🞏 Nhân viên tại các đơn vị cung cấp dịch vụ du lịch đã nghỉ hưu

Anh chị vui lòng đọc các phát biểu sau đây và đánh dấu chéo (X) vào ô thể hiện tác động của các nhân tố đến tiềm năng phát triển du lịch sinh thái tại huyện Mộc Châu, tỉnh Sơn La. Trong đó, mức độ đánh giá từ 1-7 tương đương với: 1 – Rất thấp, 2 – Thấp, 3 – Tương đối thấp, 4 – Trung bình, 5 – Tương đối cao, 6 – Cao, 7 – Rất cao.

| **Câu hỏi** | **Phát biểu** | **1** | **2** | **3** | **4** | **5** | **6** | **7** |
| --- | --- | --- | --- | --- | --- | --- | --- | --- |
| *Anh/chị đánh giá như thế nào về tác động của phong cảnh và tự nhiên đến tiềm năng thu hút du lịch của Mộc Châu?* | | | | | | | | |
| Q1 | Phong cảnh đẹp, hấp dẫn |  |  |  |  |  |  |  |
| Q2 | Khí hậu, thời tiết thuận lợi |  |  |  |  |  |  |  |
| Q3 | Hệ động thực vật phong phú |  |  |  |  |  |  |  |
| Q4 | Tính bền vững cao |  |  |  |  |  |  |  |
| Q5 | Môi trường trong lành |  |  |  |  |  |  |  |
| *Anh/chị đánh giá như thế nào về khả năng cung cấp dịch vụ du lịch của Mộc Châu?* | | | | | | | | |
| Q6 | Có chiến lược phát triển sản phẩm tốt |  |  |  |  |  |  |  |
| Q7 | Cơ sở lưu trú đa dạng, thuận lợi |  |  |  |  |  |  |  |
| Q8 | Cơ sở ăn uống đa dạng |  |  |  |  |  |  |  |
| Q9 | Mua sắm đa dạng, độc đáo |  |  |  |  |  |  |  |
| *Anh/chị đánh giá như thế nào về tác động của văn hóa bản địa đến tiềm năng thu hút du lịch của Mộc Châu?* | | | | | | | | |
| Q10 | Lịch sử tộc người độc đáo |  |  |  |  |  |  |  |
| Q11 | Ẩm thực, lễ hội đa dạng, đặc sắc |  |  |  |  |  |  |  |
| Q12 | Trang phục, kiến trúc đẹp, hấp dẫn |  |  |  |  |  |  |  |
| Q13 | Nghề truyền thống đặc sắc |  |  |  |  |  |  |  |
| Q14 | Tri thức bản địa độc đáo |  |  |  |  |  |  |  |
| Q15 | Sản phẩm du lịch đặc sắc |  |  |  |  |  |  |  |
| *Anh/chị đánh giá như thế nào về chất lượng và hiệu quả của du lịch sinh thái tại Mộc Châu?* | | | | | | | | |
| Q16 | Nhiều địa điểm, nội dung tham quan |  |  |  |  |  |  |  |
| Q17 | Sản phẩm phù hợp thị hiếu của khách |  |  |  |  |  |  |  |
| Q18 | Người dân thân thiện, hiếu khách |  |  |  |  |  |  |  |
| Q19 | Người dân tham gia hướng dẫn du lịch |  |  |  |  |  |  |  |
| Q20 | Người dân tạo lập sản phẩm du lịch |  |  |  |  |  |  |  |
| Q21 | Thông tin đầy đủ, chính xác, cập nhật |  |  |  |  |  |  |  |
| Q22 | Tiếp cận dễ dàng, thuận lợi |  |  |  |  |  |  |  |
| Q23 | Kết nối với điểm, tuyến du lịch thuận lợi |  |  |  |  |  |  |  |
| Q24 | Phương tiện di chuyển đến, đi thuận lợi |  |  |  |  |  |  |  |
| Q25 | Khung thời gian di chuyển đến linh hoạt |  |  |  |  |  |  |  |
| Q26 | *Anh/chị đánh giá như thế nào về chất lượng và hiệu quả của du lịch sinh thái tại Mộc Châu?* |  |  |  |  |  |  |  |

**XIN CHÂN THÀNH CẢM ƠN VÀ CHÚC ANH/CHỊ THÀNH CÔNG !!!**

STAKEHOLDER SURVEY FORM

**INVESTIGATION OF POTENTIAL ECO-TOURISM DEVELOPMENT
IN MOC CHAU DISTRICT, SON LA PROVINCE**

**Dear participants,**

To investigate the potential eco-tourism development in Moc Chau District (Son La province), we would like to invite you to express your views about the below statement. Your opinions provide scientific materials only for research and publications conducted by researchers and students in Tay Bac University, which advocates tourism investment and development in Moc Chau.

Please provide your personal information as follow:

Full name: _____________________________________________ Age: ______ Gender: 🞏 Male 🞏 Female

Working status: 🞏 Students/Interns majoring in tourism
🞏 Working at tourism service supplies
🞏 Temporal unemployment/freelancer at tourism service supplies
🞏 Retired (Used to work at tourism service supplies)

Please read the statements below and choose the most appropriate answers for your assessment of potential eco-tourism development in Moc Chau, Son La province. The scale for assessment ranges as follows: 1 – Very Low, 2 – Low, 3 – Relatively low, 4 – Moderate, 5 – Relatively high, 6 – High, 7 – Very High.

| **Question** | **Statement** | **1** | **2** | **3** | **4** | **5** | **6** | **7** |
| --- | --- | --- | --- | --- | --- | --- | --- | --- |
| *What do you think about the potential contribution of natural scenic landscape on eco-tourism attraction in Moc Chau District?* | | | | | | | | |
| Q1 | Breathtaking scenery and natural attractions |  |  |  |  |  |  |  |
| Q2 | Pleasant weather |  |  |  |  |  |  |  |
| Q3 | Scenic mountain and valleys |  |  |  |  |  |  |  |
| Q4 | Actions for promoting nature conservation |  |  |  |  |  |  |  |
| Q5 | Clean and tidy environment |  |  |  |  |  |  |  |
| *What do you think about available tourism service supplies in Moc Chau District?* | | | | | | | | |
| Q6 | Ecotourism activities for constructing environmental awareness and respect |  |  |  |  |  |  |  |
| Q7 | Wide choice of accommodations |  |  |  |  |  |  |  |
| Q8 | Wide selection of restaurants/cuisine |  |  |  |  |  |  |  |
| Q9 | Wide variety of shop facilities |  |  |  |  |  |  |  |
| *What do you think about the potential contribution of native ethnic culture on eco-tourism attraction in Moc Chau District?* | | | | | | | | |
| Q10 | Distinctive history and heritage |  |  |  |  |  |  |  |
| Q11 | Variety of special events/festivals |  |  |  |  |  |  |  |
| Q12 | Beautiful costumes and architectures |  |  |  |  |  |  |  |
| Q13 | Traditional handicraft |  |  |  |  |  |  |  |
| Q14 | Indigenous knowledge |  |  |  |  |  |  |  |
| Q15 | Local tourism products |  |  |  |  |  |  |  |
| *What do you think about the quality of available tourism services in Moc Chau District?* | | | | | | | | |
| Q16 | Diversity of tourism sites |  |  |  |  |  |  |  |
| Q17 | Friendliness of service |  |  |  |  |  |  |  |
| Q18 | Friendly and helpful local people |  |  |  |  |  |  |  |
| Q19 | Local tour guides |  |  |  |  |  |  |  |
| Q20 | Local participants in tourism activities |  |  |  |  |  |  |  |
| Q21 | Availability of travel information |  |  |  |  |  |  |  |
| Q22 | Convenient accessibility |  |  |  |  |  |  |  |
| Q23 | Helpfulness of welcome center |  |  |  |  |  |  |  |
| Q24 | Convenience of local transportation |  |  |  |  |  |  |  |
| Q25 | Well communicated traffic flow |  |  |  |  |  |  |  |
| Q26 | *What do you think about the potential eco-tourism development in Moc Chau District?* |  |  |  |  |  |  |  |

Thanks for your cooperation!
